# Nutritional status and dietary intake of urban residents in Gondar, Northwest Ethiopia

**DOI:** 10.1186/1471-2458-12-752

**Published:** 2012-09-07

**Authors:** Bemnet Amare, Beyene Moges, Feleke Moges, Bereket Fantahun, Mengesha Admassu, Andargachew Mulu, Afework Kassu

**Affiliations:** 1 Department of Medical Biochemistry, College of Medicine and Health Sciences, University of Gondar, P.O. Box 196, Gondar, Ethiopia; 2 Department of Immunology and Molecular Biology, College of Medicine and Health Sciences, University of Gondar, P.O. Box 196, Gondar, Ethiopia; 3 Department of Microbiology, College of Medicine and Health Sciences, University of Gondar, P.O. Box 196, Gondar, Ethiopia; 4 Department of Pediatrics, Addis Ababa University, Addis Ababa, Ethiopia; 5 Department of Environmental Health, Institute of Public Health, College of Medicine and Health Sciences, University of Gondar, P.O. Box 196, Gondar, Ethiopia

**Keywords:** Urban Ethiopia, Dietary intake, Nutritional status

## Abstract

**Background:**

There is paucity of data on the dietary intake and nutritional status of urban Ethiopians which necessitates comprehensive nutritional assessments. Therefore, the present study was aimed at evaluating the dietary intake and nutritional status of urban residents in Northwest Ethiopia.

**Methods:**

This cross-sectional community based nutrition survey was conducted by involving 356 participants (71.3% female and 28.7% male with mean age of 37.3 years). Subjects were selected by random sampling. Socio demographic data was collected by questionnaire. Height, weight, hip circumference and waist circumference were measured following standard procedures. Dietary intake was assessed by a food frequency questionnaire and 24-h dietary recall. The recommended dietary allowance was taken as the cut-off point for the assessment of the adequacy of individual nutrient intake.

**Results:**

Undernourished, overweight and obese subjects composed 12.9%, 21.3% and 5.9% of the participants, respectively. Men were taller, heavier and had higher waist to hip ratio compared to women (P < 0.05). Fish, fruits and vegetables were consumed less frequently or never at all by a large proportion of the subjects. Oil and butter were eaten daily by most of the participants. Mean energy intakes fell below the estimated energy requirements in women (1929 vs 2031 kcal/day, P = 0.05) while it was significantly higher in men participants (3001 vs 2510 kcal/day, P = 0.007). Protein intake was inadequate (<0.8 g/kg/day) in 11.2% of the participants whereas only 2.8% reported carbohydrate intake below the recommended dietary allowances (130 g/day). Inadequate intakes of calcium, retinol, thiamin, riboflavin, niacin and ascorbic acid were seen in 90.4%, 100%, 73%, 92.4%, 86.2% and 95.5% of the participants.

**Conclusions:**

The overall risk of nutritional inadequacy among the study participants was high along with their poor dietary intake. Hence, more stress should be made on planning and implementing nutritional programmes in urban settings aimed at preventing or correcting micronutrient and some macronutrient deficiencies which may be useful in preventing nutrition related diseases in life.

## Background

Nutrition is an important factor in health and disease [1]. The nutrition transition is marked by a shift away from relatively monotonous diets of varying nutritional quality toward an industrialized diet that is usually more varied, includes more preprocessed food, more food of animal origin, more added sugar and fat, and often more alcohol. This is accompanied by shift in the structure of occupations and leisure toward reduced physical activity [2].

The pattern of nutritional disorders in the developing world is further complicated by sociological changes which are taking place due to urbanization and changing lifestyles [3,4]. In five out of the six regions of WHO deaths caused by chronic diseases dominate the mortality statistics [5,6]. Although infectious diseases, still predominate in sub-Saharan Africa and will do so for the foreseeable future, 79% of all deaths worldwide that are attributable to chronic diseases are already occurring in developing countries [5,6].

Epidemiological studies show that nutritional inadequacy can influence the incidence and the severity of infectious diseases [7-10]. In Ethiopia, nutritional problems and infectious diseases are amongst the major health problems [8]. Chronic health disorders such as obesity, diabetes and cardiovascular diseases (CVDs) have been increasing in the country since the last few decades [9]. According to the Ethiopian nationwide study on income, expenditure and consumption of 2005, fruits accounted for the lowest proportion (0.2%) of the per capita expenditure as compared to cereals (20.4%), pulses (3.9%), oils and fats (2%), khat (1.4%), or alcohol and tobacco (1.1%). A strong association between nutritional impairment and the development of chronic diseases such as cardiovascular diseases, cancer, and diabetes has been reported. Population-based data on cause of death from a few isolated studies, in predominantly rural populations, in Ethiopia demonstrate that a considerable proportion of the disease burden in these populations is due to CVD and other chronic diseases [11].

However, there is paucity of data on dietary intakes and nutritional status in Northwest Ethiopia. Therefore, this study was aimed to evaluate the dietary intake and anthropometric variables of urban residents in Northwest Ethiopia [12].

## Methods

### Study area and subjects

This cross-sectional study was conducted in Gondar city, Northwest Ethiopia in July 2005. Gondar is a zonal capital city located 750kms north of Addis Ababa in Amhara Region. The city has a longitude and latitude of 12°36′N 37°28′E. Based on figures from the Ethiopian Central Statistical Agency in 2005, Gondar has an estimated total population of 194,773 of whom 97,625 were males and 97,148 were females. Sample size was calculated based on expected estimates of 50% of BMI < 18.5, 95% confidence limits, and a 5% marginal error, the required sample was 384. Probability sampling in a form of simple random and two-stage probability sampling method was used for selecting the required size. The first stage of the sampling was started by selecting kebeles (smallest administrative unit) using simple random sampling. At the second stage, a random sample of households was selected based on a sampling frame from the 1994 census and adapted for recent population changes.

Out of 384 participants, data of 28(7%) of the study participants were incomplete and excluded of the statistical analysis. Nutritional status and dietary intake indicators was primary variables of interest. In addition, a structured questionnaire was used to collect information on socio-demographic variables including sex, age, religion, marital status, occupation, educational status and monthly family income. Monthly family income was estimated by combining incomes reported for husband, wife, son and/or daughter. The inclusion criteria for participation were age >18 year, not acutely ill at the time of survey and not diagnosed for chronic illnesses. Ethical approval for this study was obtained from the Research Ethics Committee of the University of Gondar. Informed consent was obtained from all subjects.

### Anthropometric and body composition measurements

Body weight (kg) was measured using an electronic scale to the nearest 10 g, and standing height was measured using a wall stadiometer to the nearest 0.1 cm. Subjects were instructed to take off their shoes before performing these measurements. Body Mass Index (BMI) was calculated as body weight (kg)/height (m^2^). The classifications of BMI applied in this study were recommended by the World Health Organization (WHO) [13] BMI values of <18.5 kg/m^2^ and >25 kg/m^2^ represented thinness and overweight, respectively. An acceptable weight was considered to fall within these two extremes. Waist and hip circumferences were measured with a flexible steel metric tape at the nearest 0.5 cm. Central obesity was also calculated and defined on the basis of WHR. The cut-off value of central obesity was considered high risk WHR= >0.80 or waist measurement >80% of hip measurement for women for females and >0.95 for males that is >95% for men indicates central (upper body) obesity and is considered high risk for diabetes & CVS disorders. A WHR below these cut-off levels is considered low risk [13].

### Interview using food frequency questionnaire

Data were collected by face-to-face interview using a structured Food Frequency Questionnaire (FFQ) modified from the Helen Keller International FFQ that was used previously in Ethiopia, to estimate meat and vegetable consumption that was in addition to the staple food intake [14]. The FFQ included eight food categories (Meat, Egg, Fish, Fat rich food, Vegetables, Fruits, Diary products, Sweet food) and was designed to obtain qualitative information about the usual food consumption patterns with an aim to assess the frequency with which certain food items or groups are consumed during a specific time period [15]. All frequency variables were coded as never or hardly ever, once a month, 2–3 times a month, once a week, 2–3 times a week, 4–6 times a week, and at least once a day.

### 24-h dietary recall

The respondents were asked to recall the exact food intake of the previous day. Detailed descriptions of all foods including recipes and beverages consumed were recorded. Quantities of food consumed were estimated in household measures. One single 24-h recall was collected for every participant. Only one adult individual was selected from a house hold. For the transformation of household measurements and centimetres into grams, the portion sizes were weighed with a digital household dietary scale (Omron Electronic kitchen scale, Omron, Tokyo, Japan). Information from the 24-h protocols was entered and analyzed with Microsoft EXCEL software. The various food items mentioned in the recall were transformed into their corresponding weight of raw food ingredients. Ethiopian food composition tables [16] or food composition table for use in Africa [17], for those not available in the former, was used to calculate energy and nutrients content. Major nutrients in the food composition tables were measured. The data were subsequently converted into the amount of energy and nutrient intake per individual per day. Relative validity of 24-h recall was determined by comparison data obtained from the same participants using a food-frequency questionnaire. Furthermore, three 24-h recalls were repeated in 10% of the sample. The dietary results are under preparation.

Adequacy of the macronutrients and micronutrients intake was evaluated according to the Dietary Reference Intakes (DRI) of The Institute of Medicine of The National Academies [18]. The reported energy intakes were compared with estimated minimal energy requirements to assess adequacy. Basal Metabolic Rate (BMR) was estimated using the sex and age specific equations of FAO/WHO/UNU expert consultations. The BMR was then multiplied by a factor which stands for physical activity level for each individual [19].

### Dietary quality

As a measure of overall nutrient adequacy, mean adequacy ratio (MAR) was calculated as the mean of the nutrient adequacy ratios (NARs) for the intake of energy and nine nutrients (protein, calcium, iron, phosphorus, retinol, thiamin, riboflavin, niacin, ascorbic acid), each truncated at 1 so that a nutrient with a high NAR could not compensate for a nutrient with a low NAR [20].

### Statistical analysis

The mean ± SD daily nutrient intake was computed and tabulated. The mean intakes of energy, macronutrients and micronutrients were compared between men and women by independent sample *t*-test. Chi square test of proportion was used to determine the percentage of participants with intakes at or below the recommended daily allowance and adequate intakes. Correlation test was tested to examine the relationship between socioeconomic factors on dietary intake and selected nutritional variables. All statistical analyses were undertaken using SPSS version 13. P values less than 0.05 were considered statistically significant.

## Results

### Socio-demographic and anthropometric profile

Of the 384 study participants, 356 were studied (93% response rate). Of the 28 excluded, 8 were absent on the day of the interview, 5 adults refused on behalf of their household and data for the rest was not complete. The details of socio-demographic characteristics of the study participants are presented in Table1. Of the 356 participants, 255 (71.3%) were females and 101 (28.7%) were males. Their mean age was 37.3 years (SD = 13.1; range 18 – 80 years). A substantial majority of them were Christians (90.2%) and married (57%). About 14% of the sample had no formal education. An additional 26.1% had received some years of primary school education, whereas only 16.8% had tertiary level training. The majority of males (54.5%) were government employees while that of females were house wives (37.6%). Twenty four percent of the sample lived with a monthly income of less than 30 US dollar and additional 28% lived for less than 60 US dollar per month.

**Table 1:** Socio-demographic profile of subjects included in nutrition survey, Gondar, Ethiopia, 2005

**Parameter**	**Total**	**Male**	**Female**
	**(n = 356)**	**(n = 101)**	**(n = 255)**
Age in year			
18–24	61 (17.1)	13 (12.9)	48 (18.8)
25–34	100 (28.1)	23 (22.8)	77 (30.2)
35–44	95 (26.7)	25 (24.8)	70 (27.5)
45–54	57 (16.0)	21 (20.8)	36 (14.1)
55–64	29 (8.1)	12 (11.9)	17 (6.7)
>65	14 (3.9)	7 (6.9)	7 (2.7)
Religion			
Christian	321 (90.2)	90 (89.1)	231 (90.6)
Muslim	31 (8.7)	10 (9.9)	21 (8.2)
Other	4 (1.1)	1 (1.0)	3 (1.2)
Marital status			
Married	203 (57.0)	78 (77.2)	125 (49.0)
Never married	86 (24.2)	20 (19.8)	66 (25.9)
Divorced, widowed, separated	67 (18.8)	3 (3.0)	64 (25.1)
Occupation			
House wife	96 (27.0)	-	96 (37.6)
Government employee	114 (32.0)	55 (54.5)	59 (23.1)
Daily laborer	44 (12.4)	15 (14.9)	29 (11.4)
Other	102 (28.7)	31 (30.7)	71 (27.8)
Educational status			
No	49 (13.8)	3 (3.0)	46 (18.0)
Primary	93 (26.1)	23 (22.8)	70 (27.5)
Secondary	154 (43.3)	37 (36.6)	117 (45.9)
Tertiary	60 (16.8)	38 (37.6)	22 (8.6)
Monthly income in Birr			
Mean ± SD	663.9 ± 522.8	807.1 ± 568.6	607.2 ± 493.4
Range	20.0 – 2872.0	60.0-2500.0	20.0-2872.0
<250	86 (24.2)		
251–500	101 (28.4)		
501–1000	100 (28.1)		
>1000	69 (19.4)		

Mean anthropometrical measures are presented in Table2. Men were taller, heavier and had higher waist to hip ratio compared to women (P < 0.05). Overweight subjects composed 21.3% of the total sample population, whereas obese subjects composed 5.9% of the above sample. Among females, 19.6% were overweight and 7.1% were obese, whereas among males, 25.7% were overweight and only 3% were obese. The cutoff points used for classification of participants as overweight and obese were similar to those introduced by the WHO [14]. Waist circumference was higher in males than females (P < 0.05) but hip circumference was higher in females (Table2). 

**Table 2:** Anthropometric status of study participants subjects included in nutrition survey, Gondar, Ethiopia, 2005

**Parameter**	**Total**	**Male**	**Female**	**P-value**
	**(n = 356)**	**(n = 101)**	**(n = 255)**	
Weight in kilogram				
Mean ± SD	58.9 ± 11.2	65.6 ± 10.5	56.3 ± 10.5	<0.001
Median (Range)	58.1 (35.0 – 94.7)	65 (42–93.9)	55 (35–94.7)	
Height in meter				
Mean ± SD	1.61 ± 0.08	1.69 ± 0.07	1.57 ± 0.06	<0.001
Median (Range)	1.60 (1.20.0-1.86)	1.70 (1.47-1.86)	1.58 (1.20-1.70)	
BMI				
Mean ± SD	22.8 ± 3.9	22.9 ± 3.5	22.8 ± 4.1	0.8
Median (range)	22.5 (15.2 – 38.4)	22.7 (16.3-36.2)	22.3 (15.2-38.4)	
<18.5	46 (12.9)	9 (8.9)	37 (14.5)	
18.5-24.9	213 (59.8)	63 (62.4)	150 (58.8)	
25–29.9	76 (21.3)	26 (25.7)	50 (19.6)	
30+	21 (5.9)	3 (3.0)	18 (7.1)	
Hip circumference				
Mean ± SD	97.0 ± 11.6	96.4 ± 10.0	97.3 ± 12.3	0.5
Median (Range)	97.0 (61 – 132)	96.5 (68–125)	98 (61–132)	
Waist circumference				
Mean ± SD	85.7 ± 13.6	88.4 ± 14.7	84.6 ± 13.0	0.01
Median (Range)	85.0 (52 – 152)	86 (57–152.4)	85 (52–123)	
W/H ratio				
Mean ± SD	0.89 ± 0.11	0.92 ± 0.13	0.87 ± 0.11	0.001
Median (Range)	0.87 (0.62-1.59)	0.91 (0.62-1.54)	0.86 (0.63-1.59)	

### Food consumption and frequency

Table3 compares mean daily intakes of food in men and women. Mean overall consumption of Fish, fruits and vegetables, separately and totally, was lower in large proportion of the subjects. Oil and butter was eaten daily by most subjects *(n* = 310). Meat, fish, sweets, milk and yoghurt were consumed in significantly higher amounts by women (P < 0.05). However, although not statistically significant, men's mean consumption of oil and butter was higher than women's.

**Table 3:** Comparison of mean food intake (g/day) of women and men in Gondar, Ethiopia, 2005

**Food**	**Women**	**Men**	**Difference between**	
	**(n = 255)**	**(n = 101)**	**men and women**	**p-value**
Meat	^a^ 3.39 ± 1.50	2.72 ± 1.65	0.67839	0.000
Eggs	2.47 ± 1.78	2.18 ± 1.64	0.28888	NS
Fish	0.33 ± 0.75	0.13 ± 0.49	0.19732	0.004
Oil and butter	5.52 ± 1.36	5.64 ± 1.21	−0.12436	NS
Vegetables	2.82 ± 1.56	2.76 ± 1.58	0.06100	NS
Fruits	2.22 ± 1.85	1.76 ± 1.64	0.45312	0.024
Sweets	1.84 ± 2.21	1.16 ± 1.83	0.68472	0.003
Milk and yogurt	3.60 ± 2.18	2.42 ± 2.11	1.18435	0.000

^a^Results are expressed as the mean ± SD of the participants consuming different frequencies of each food items in the study period. NS: not significant.

Table4 shows the average daily per capita consumption of the various food items by the sample population in comparison to previous data from a national survey. More than half of respondents reported intake of energy-dense food and alcohol. One fourth of men (25/101) and 6.6% (17/255) women reported having consumed alcoholic beverages (beer, *tela* or *katikala*) during the previous day. Only two men reported to have consumed *katikala* (home brewed liquor). Beer consumption was reported by 10 women and 22 men (mean intake 1047 ml and 762 ml, respectively) while *tela* consumption was reported by four men (mean 488 ml) and seven women (mean 779 ml).

**Table 4:** Daily food consumption per capita (gram/day) in Gondar, Ethiopia in 2005 compared to 1982 reports

**Food group/item**	**Present study**	**1981-1982 **[15]
Grain	391 gm	360gm
Vegetable oil & butter	33	
Vegetable oil	29	13
Butter	4	
Root and tubers	94	64
Other vegetables	10	26
Fruit	0.8	13
Salt	6	
Sugar	21	
Chili	15	
Meat	52	32
Milk	17	0.05
Egg	7	

### Macronutrient intake

Mean energy, macronutrient and fiber intakes of the study subjects and comparison of percentage contribution to total energy from macronutrients is presented in Table5. Mean energy intakes was significantly higher in men participants (3001 vs 2510 kcal/day, P = 0.007). However, the mean energy intake for both men and women was not significantly different from the estimated mean energy requirement (2234 vs 2167, P = 0.3). About 45.5% (162/356) of the participants had reported energy intake within 80-120% of the estimated requirement while 18.0% (64/356) reported its intake above 120% of the estimated requirement. Reported energy intake was higher than estimated energy requirement in 122 study participants. The mean fat, protein and carbohydrate intake (g/day) was 80, 79 and 320 and their percentage contribution for total energy was 33.0%, 14.1% and 52.9%, respectively. Men had significantly higher energy and macronutrient intake than women (P < 0.001). Protein intake was inadequate (<0.8 g/kg/day) in 11.2% (40/356) of the participants. Only 2.8% (10/356) reported carbohydrate intake below the Recommended Dietary Allowances (RDA) (130 g/day). About a third (31.7%, 13/356) of the study subjects had fat intake which contributed for less than 30% of total energy per day. Mean dietary fiber intake (19 g/day) did not meet the prudent dietary recommendation (38 g/day for men and 25 g/day for women).

**Table 5:** Distribution of energy, macronutrient and fiber intakes of men and women in Gondar city, Ethiopia 2005

**Intake per day**	RDA**	** All**	** Men**	** Women**	**Difference between men and women**
	**Men, women**				
Energy (kcal)*	3067, 2403	^a^2233.89 ± 1261.56	3001.29 ± 1780.52	1929.95 ± 805.81	0.000
		^b^1914.45 (661.25-7670.41)	2247.58 (989.43-7670.41)	1802.86 (661.25-6279.73)	
Protein (g)					
% energy from protein	56, 46	79.23 ± 36.68	104.03 ± 48.68	69.41 ± 24.63	0.000
		71.26 (9.67-262.84)	85.83 (38.32-262.84)	67.05 (9.68-178.59)	
	^c^10-15%	13.71	13.48	13.82	
Carbohydrate (g)	130	320.29 ± 246.78	460.42 ± 366.16	264.78 ± 146.41	0.000
		255.91 (100.55-1421.03)	292.23 (113.87-1421.03)	233.67 (100.55-1174.65)	
% energy from carbohydrate	^c^55-75%	55.35	59.67	52.73	
Fat (g)	-	79.57 ± 31.94	92.06 ± 33.60	74.63 ± 29.91	0.000
		73.82 (21.24-231.45)	86.23 (39.71-231.45)	71.13 (21.24-193.19)	
% energy from fat	^c^15-30%	30.94	26.85	33.44	
Dietary fiber (g)	38, 25	19.27 ± 7.07	21.44 ± 7.43	18.41 ± 6.75	0.000
		18.63 (1.59-44.63)	20.31 (8.52-44.63)	18.15 (1.59-44.41)	

a: mean ± SD, b: median (range), c: Ranges of population energy intake goal as % of total energy, *: estimated energy requirement.

**: recommended dietary allowances.

### Micronutrient intake

Average intake of minerals and vitamins of the subjects and prevalence of inadequate micronutrient intakes, that were computed based on RDA reference values, are presented in Table6. Inadequate intakes of calcium, retinol, thiamin, riboflavin, niacin and ascorbic acid were seen in 90.4%, 100%, 73%, 92.4%, 86.2% and 95.5% of the participants whereas intakes of iron and phosphorus were found to be adequate except in a few subjects 0.3% and 1.4%, respectively (Table7). Mean MAR was 0.74 for the total sample. A diet that covers the recommended intake for all nutrients has a MAR of 1 and a MAR below one indicates lower than the recommended intake for one or more nutrients [21]. A significantly higher proportion of women were deficient in calcium, thiamin and niacin compared to men while the proportion of inadequate retinol, riboflavin and ascorbic acid intakes were similar between the two sexes. 

**Table 6:** Distribution of mean micronutrient intake of men and women in Gondar, Ethiopia 2005

**Nutrient**	RDA**	**All**	**Men**	**Women**	**Difference between men and women**
	**Men, women**	**(n = 356)**	**(n = 101)**	**(n = 255)**	
Calcium (mg)	1000	^a^663.53 ± 271.04	808.62 ± 323.71	606.07 ± 223.00	0.000
		^b^613.56 (39.20-2471.10)	736.53 (189.90-2471.10)	563.65 (39.20-1426.01)	
		^c^90.4	82 (81.2)	240 (94.1)	
Phosphorus (mg)	700	1708.60 ± 1035.61	2340.67 ± 1487.01	1458.25 ± 637.66	0.000
		1436.15 (211.80-6542.52)	1736.89 (836.76-6542.52)	1327.42 (211.80-4875.87)	
		5 (1.4)	0 (0.0)	5 (2.0)	
Iron (mg)	8, 18	109.29 ± 68.94	138.27 ± 89.99	97.81 ± 54.65	0.000
		98.87 (4.08-879.31)	123.77 (27.79-879.31)	85.91 (4.08-345.03)	
		1 (0.3)	0 (0.0)	1 (0.4)	
Retinol (ug)	900, 700	22.75 ± 78.79	32.19 ± 102.97	19.01 ± 66.72	0.15
		0 (0–560)	0 (0–560)	0 (0–438)	
		100 (100.0)	101 (100.0)	255 (100.0)	
B-carotene (ug)	-	226.20 ± 225.81	290.01 ± 277.53	200.93 ± 196.68	0.001
		159.00 (0–2332)	243.25 (0–2332)	137.80 (0–1168)	
Thiamin (mg)	1.2, 1.1	1.22 ± 1.06	1.81 ± 1.60	0.98 ± 0.61	0.000
		0.93 (.15-6.21)	1.11 (.39-6.22)	0.84 (.15-4.77)	
		260 (73.0)	60 (59.4)	200 (78.4)	
Riboflavin (mg)	1.3, 1.1	0.73 ± 0.30	0.84 ± .35	0.68 ± 0.26	0.000
		0.69 (.02-2.98)	0.79 (.05-2.98)	0.65 (.02-1.97)	
		329 (92.4)	91 (90.1)	238 (93.3)	
Niacin (mg)	16, 14	15.03 ± 20.31	26.28 ± 31.34	10.57 ± 10.94	0.000
		8.87 (1.33-114.09)	11.19 (3.74-114.09)	8.29 (1.33-80.58)	
		301 (86.2)	71 (70.3)	236 (92.5)	
Ascorbic acid (mg)	90, 75	24.10 ± 26.99	28.45 ± 28.00	22.38 ± 26.44	0.056
		15.17 (0–206.4)	20.90 (0.1-139.0)	14.64 (0–206.4)	
		340 (95.5)	96 (95.0)	244 (95.7)	

a: mean ± SD, b: median (range), c: number (proportion) of subject with inadequate intake.

**: recommended dietary allowances.

**Table 7:** Description of mean adequacy ratio (MAR) and nutrient adequacy ratios (NAR) calculated from FFQ (Ethiopia, 2005)

	**mean ± SD**	**% below recommended**
		**nutrient intake compared to RDA**
NAR* energy	0.81 ± 0.53	
NAR protein	1.53 ± 0.82	11.2%
NAR calcium	0.66 ± 0.27	90.4%
NAR iron	10.94 ± 6.68	0.3%
NAR Phosphorous	2.44 ± 1.48	1.4%
NAR Retinol	0.03 ± 0.10	100%
NAR Thiamin	1.15 ± 0.96	73%
NAR Riboflavin	0.59 ± 0.27	92.4%
NAR Niacin	1.01 ± 1.43	86.2%
NAR Ascorbic acid	0.38 ± 0.32	95.5%
MAR**	0.74 ± 0.10	

*: nutrient adequacy ratio, **: mean adequacy ratio, : recommended dietary allowances.

The correlation between socioeconomic variables and frequency of food consumption was tested. Income at the time of data collection had a positive correlation with BMI and level of education (p < 0.01). In addition, the correlation between income and frequency of consumption for most of the foods were significantly positive (p < 0.01). The correlation coefficients between BMI and the food consumption frequency were significantly positive for meat (r = 0.36; p < 0.01), egg (r = 0.177; p < 0.01), vegetables (r = 0.252; p < 0.01), fruits (r = 0.263; p < 0.01), sweets (r = 0.124; p < 0.05) and Milk (r = 0.217; p < 0.01). Similarly, the association between level of education and food consumption frequency was positive, except for oil and butter (r = −0.082) (Table8).

**Table 8:** Correlation coefficients for changes in frequency of food consumption and socio-economic variables among men and women in Gondar city, Ethiopia 2005

	**Income**	**BMI**	**Level of education**
Income	**-**	**-**	**-**
BMI	0.379**	**-**	**-**
Level of education	0.538**	0.168**	**-**
Meat	0.558**	0.366**	0.475**
Egg	0.409**	0.177**	0.410**
Fish	0.249**	0.080	0.307**
Oil and butter	0.047	0.058	−0.082
Vegetables	0.419**	0.252**	0.352**
Fruit	0.534**	0.263**	0.470**
Sweets	0.436**	0.124*	0.440**
Milk and yogurt	0.551**	0.217**	0.522**

** Correlation is significant at the 0.01 level (2-tailed).

* Correlation is significant at the 0.05 level (2-tailed).

## Discussion

This cross-sectional study provides data on the nutritional status and dietary intake of urban residents in Gondar city, Northwest Ethiopia. The results of this study indicate that the diets of urban residents included in this study are undesirable according to the Dietary Reference Intakes (DRIs) used. Overall, participant diets included too much energy-dense food and saturated fat and inadequate intakes of micronutrients. The men seem to have more than adequate intake compared to women. Irrespective of sex, micronutrient intake is very low in the area. BMI data point out the prevalence of a high percentage of overweight and obese subjects in both sexes.

The results also showed that males had a greater mean in BMI and Waist-to-Hip Ratio (WHR) than females, related to physiological differences between male and females [22,23]. Higher BMI and WHR may be considered as indicators of high risk factors for cardiovascular disease since they have strong relation to lipid profile in both sex groups [22,24-26]. A considerable proportion of urban residents (21.3%) in Gondar had overweight and obesity in contrast to previous reports of low prevalence of overweight in Ethiopia [27]. Increased dietary energy and fat intake, coupled with insufficient physical activity, is implicated in the rapidly growing prevalence of overweight and obesity in sub Saharan Africa, where there is a longstanding tradition favoring obesity over thinness. Overweight in general, and abdominal obesity in men, is regarded as a sign of health and wealth in many communities in Africa, including Ethiopia. Thinness, in contrast, is considered as a sign of illness or poverty [25,26].

Although, there is limited data on the BMI distribution or prevalence of overweight and obesity in sub Saharan African countries, in other African countries, the prevalence of obesity was consistently higher in urban areas [24,25].

Although, eating more vegetables and fruits as the part of Dietary Approaches to Stop Hypertension (DASH) diet are associated with reduced risk for cardiovascular diseases [28] In Gondar and most cities in the country, people are reluctant to consume vegetables especially in commercial food catering places and in social occasions where food is served to large number of guests. There is widespread fear of infection, particularly with amoeba, from consuming uncooked vegetables. It is common to see that a large part of the vegetables cultivated in cities are contaminated with water that is contaminated with sewerage and use of infected manure as a fertilizer.

Fruits are not also part of the regular daily diet in Ethiopia. Unlike other populations where fruits follow meals for dessert, instead tea and coffee are the predominant accessories to meals in this population. Fruits are more commonly consumed during weekends, social occasions or holidays. They are the preferred gift while visiting sick people (patients) at home or in health facilities. The price of common fruits, such as oranges and bananas, has remained generally low for many years in Ethiopia until a recent surge, which was partly attributed to increasing exports. In addition, according to results of this study, consumption of fish is very small due to cultural aversion to eating fish although one of the biggest lakes (Lake Tana) is only 60 km from Gondar.

Intake of fat by the study participants was higher than the suggested acceptable macronutrient distribution range which is a negative impact of nutrition transition [29-31]. The dietary changes of the nutrition transition involve large increases in the consumption of fat (especially saturated fat) and sugar, marked increases in animal products, and a decline in unrefined cereal and, thus, in fiber intakes [32,33]. It is recommended that fiber intake could be improved by taking whole grain than refined grain intake; thus, nutrition education programs are needed to improve the dietary intake and for healthy eating pattern [34]. As in many sub-Saharan Africa countries, in Ethiopia, an increased level of body fat is associated with beauty, prosperity, health, and prestige, despite its negative impact on health. Thinness, in contrast, is perceived to be a sign of ill health or poverty and is something to be feared and avoided, particularly in recent years, when it has been associated with AIDS [26,35].

Micronutrients are required for virtually all metabolic and developmental processes. The large percentage of study subjects with inadequate intakes of calcium, retinol, thiamin, riboflavin, niacin and ascorbic acid indicates that micronutrient deficiencies are still major public health problems in developing countries [36-38]. These dietary pattern changes in which the macronutrient pattern could already be associated with an increased risk of overweight, obesity and other non communicable diseases [39,40] while the improvements in micronutrient intakes in urban subjects, did not reach recommended values for some micronutrients [34,41]. It is conceivable that in many overweight and obese subjects, sub-optimal micronutrient intakes could lead to a “double burden” of co-existence of under- and over-nutrition in the same person. It is further conceivable that some of the observed micronutrient deficiencies, such as those with anti-oxidant properties, could contribute to the increased risk of non communicable diseases in these subjects.

Our data agree with previous studies in different countries suggesting lower intakes of essential nutrients, vitamins, and minerals, especially calcium, thiamin and niacin in developing countries during nutrition transition [42-44]. It is understandable that with economic development, people will choose to follow a more palatable diet than traditional diets high in fiber and low in fat. But it is more difficult to understand why adult Africans, often from poor, food-insecure households, are so vulnerable to obesity when they experience the nutrition transition. It has been suggested that based on the Barker hypothesis [45] of fetal programming for vulnerability to non communicable diseases in later life when the expectant mother is nutritionally compromised, stunted children and adults born from these mothers in African households are more vulnerable to obesity when they are suddenly following a modern, “Western” diet [46].

This study has also shown that the major determinants for frequency of food consumption among adults are socioeconomic. The more income the family generates, the better their frequency of food consumption and hence BMI. Although not statistically significant, level of education is negatively correlated with frequency of consumption for oil and butter. Health education campaigns warning against butter as source of saturated fatty acids and recommending unsaturated fats might have influenced the behaviors of the highly educated in the study area. Nutrition education of the masses needs to be intensified to encourage a healthy lifestyle. Food fortification programmes to include micronutrients are also advocated.

The limitations of this study include single 24 h dietary recall, thereby providing a less precise measure of intake. The study did not include the rural communities due to financial constraint. Yet, the representativeness of the urban population samples to the corresponding strata in the whole country is limited due to possibly marked diversity in socioeconomic and cultural background of different populations in the country. Additionally, the cross-sectional nature of our study ruled out a determination of the role of poor diet in the development of high-risk anthropometric measures or the role of lack of knowledge of nutrition in making poor dietary habit.

## Conclusions

The nutrition transition in sub-Saharan African countries is complex, because overweight, obesity and other non-communicable diseases emerged before the problems of under-nutrition and micronutrient deficiencies have been solved. According to the results of this study, it is concluded that the dietary intake and nutritional inadequacy of Northwest Ethiopia urban residents was poor, especially they do not meet the standards of adequacy for micronutrients and that it reflects the dietary intake and eating patterns observed in other urban parts of the country. However, these data must be interpreted with caution because the RDA is set at a level higher than most individuals’ requirements, individuals consuming less than the RDA may still have adequate consumption levels. It is recommended that further concerted research be undertaken in different geographic regions of the country, for a better understanding of the nutrition transition in Ethiopia and in order to design interventions that are useful in promoting healthy lifestyles and thus preventing nutrition-related diseases later in life. In addition, we also recommend constructing a database of dietary intake representative of Ethiopian population with the eventual goal of establishing population reference intakes specifically targeted to Ethiopians.

## Competing interests

The authors declare that they have no competing interests.

## Authors' contributions

AK, BA, MA, AM, BM, FM and BF were all involved in the design of the study, carrying out the data collection, and drafting the manuscript. All authors read and approved the final manuscript.

## Pre-publication history

The pre-publication history for this paper can be accessed here:

http://www.biomedcentral.com/1471-2458/12/752/prepub
